# Multisensor Estimation Fusion with Gaussian Process for Nonlinear Dynamic Systems

**DOI:** 10.3390/e21111126

**Published:** 2019-11-16

**Authors:** Yiwei Liao, Jiangqiong Xie, Zhiguo Wang, Xiaojing Shen

**Affiliations:** 1 School of Mathematics, Sichuan University, Chengdu 610064, China; lyw@stu.scu.edu.cn (Y.L.); xiejiangqiong@163.com (J.X.); 2 School of Science and Engineering, The Chinese University of Hong Kong, Shenzhen 518172, China; wangzhiguo@cuhk.edu.cn; 3 Department of Electronic Engineering and Information Science, University of Science and Technology of China, Hefei 230026, China

**Keywords:** multisensor estimation fusion, Gaussian process, nonlinear dynamic systems, data driven modeling, target tracking, information fusion

## Abstract

The Gaussian process is gaining increasing importance in different areas such as signal processing, machine learning, robotics, control and aerospace and electronic systems, since it can represent unknown system functions by posterior probability. This paper investigates multisensor fusion in the setting of Gaussian process estimation for nonlinear dynamic systems. In order to overcome the difficulty caused by the unknown nonlinear system models, we associate the transition and measurement functions with the Gaussian process regression models, then the advantages of the non-parametric feature of the Gaussian process can be fully extracted for state estimation. Next, based on the Gaussian process filters, we propose two different fusion methods, centralized estimation fusion and distributed estimation fusion, to utilize the multisensor measurement information. Furthermore, the equivalence of the two proposed fusion methods is established by rigorous analysis. Finally, numerical examples for nonlinear target tracking systems demonstrate the equivalence and show that the multisensor estimation fusion performs better than the single sensor. Meanwhile, the proposed fusion methods outperform the convex combination method and the relaxed Chebyshev center covariance intersection fusion algorithm.

## 1. Introduction

Estimation in nonlinear systems is extremely important because almost all practical systems involve nonlinearities of one kind or another [1,2], such as target tracking, vehicle navigation, automatic control and robotics [3,4]. In the case of nonlinearities, estimation cannot be obtained analytically in general. Some methods based on exact parametric models and ideas of the Kalman filter (KF) have been developed. The Extended Kalman filter (EKF) [5] was the most common application to nonlinear systems, which simply linearizes all nonlinear functions via Taylor-series expansion and substitutes Jacobian matrices for the linear transformations into the KF equations. The unscented transformation was introduced to address the deficiencies of linearization, namely unscented Kalman filters (UKF) [1], which provided a more direct and explicit mechanism for transforming mean and covariance matrices. Under the Bayesian framework, particle filter (PF) was presented by constructing the posterior probability density function of the state based on all available information in Reference [6]. However, for nonlinear systems, these methods were usually assumed that the nonlinear relationships are known. Lack of modeling accuracy, including the identification of the noise and the model parameters, was inevitable [7]. In many applications, most real dynamic systems are difficult to obtain the exact models and system noises due to the complexity of the target motion environment, therefore, the parameterized estimation methods may be invalid.

To overcome the limitations of parametric models, researchers have recently employed so called non-parametric methods like the Gaussian process [8] to learn models for dynamic systems. More specifically, the functional representation needs to be learned from the training data before the filtering prediction and update step [9]. Gaussian processes have been increasingly attracting the interests in machine learning, signal processing, robotics, control and aerospace and electronic systems [10,11,12]. For example, Gaussian process models are used as the surrogate models for complex physics models in Reference [13]. The advantages stemmed from the fact that Gaussian processes take both the noise in the system and the uncertainty in the model into consideration [14]. In the context of modeling the dynamic systems, Gaussian processes can be used as prior over the transition function and measurement function. By analyzing the correlation among given training data, Gaussian process models can provide the posterior distributions over functions through the combination of the prior and the data. For the cases that ground truth data are unavailable or can only be determined approximately, Gaussian process latent variable models were developed in References [15,16] and they were extended to the setting of dynamical robotics systems in Reference [17]. In order to reduce the cubic complexity of Gaussian process training for the fixed number of training points, sparse Gaussian processes were developed (see e.g., [18,19,20,21,22,23]). K-optimality was used to improve the stability of the Gaussian process prediction in Reference [24].

So far, Gaussian process models have been applied successfully to massive nonlinear dynamic systems. Motivated by modeling human motion, learning nonlinear dynamical models with Gaussian process was investigated in Reference [16]. Many filtering methods, such as the Gaussian process extended Kalman filters (GP-EKF) [25], the Gaussian process unscented Kalman filters (GP-UKF) [26], the Gaussian process particle filters (GP-PF) [27], GP-BayesFilters [14] and the Gaussian process assumed density filters (GP-ADF) [7,10] were derived by incorporating the Gaussian process transition model and Gaussian process measurement model into the classic filters. GP-ADF was an efficient form of assumed density filtering (ADF) introduced in References [28,29,30] and propagated the full Gaussian density [7]. Although Gaussian processes have been around for decades, they mainly focus on single sensor systems.

With the rapid development of the sensor technology, computer science, communication technology and information technology, multisensor systems are widely used in military and civil fields [31,32,33,34,35]. Benefited from the application of multiple sensors, multisensor data fusion makes more comprehensive and accurate decision by integrating the available information from multiple sensors and has attracted lots of research interests. Generally speaking, the multisensor estimation fusion mainly contains centralized estimation fusion and distributed estimation fusion. Centralized estimation fusion is that a central processor receives all measurement data from sensors without processing and uses them to estimate the state [36,37]. In general, many filtering algorithms in single sensor system can be applied to the multisensor systems, since measurement value can be stacked and regarded as one measurement. On the other hand, distributed estimation fusion has several advantages in terms of reliability, survivability, communication bandwidth and computational burdens [38,39,40,41,42], which make it desirable in real applications such as surveillance, tracking and battle management. In the distributed setting, each local sensor deals with its own measurement data and transmits the local state estimation to the fusion center for the purpose of more accurate estimation fusion. So far, a variety of distributed fusion methods have been investigated for different occasions, such as References [37,40,43,44,45,46]. A convex combination method was given to fuse the local estimates in Reference [43]. For the unavailable cross correlation matrix problem, a relaxed Chebyshev center covariance intersection (RCC-CI) algorithm was also provided to fuse the local estimates in Reference [37]. A current review of distributed multisensor data fusion under unknown correlation can be seen in Reference [47]. However, these multisensor estimation fusion methods are mainly based on the exact dynamic models or known nonlinear functions. For the cases in which accurate parametric models are difficult to obtain, it is worth considering integrating Gaussian processes with multisensor estimation fusion to improve the system’s performance.

In this paper, we focus on multisensor estimation fusion including the centralized and distributed fusion methods with Gaussian process for nonlinear dynamic systems. Firstly, given the training data, we learn the dynamic models with the Gaussian process and derive multisensor estimation fusion methods based on the Gaussian process models for nonlinear dynamic systems, which can avoid inappropriate parametric models and improve predictive ability. Combining with the single senor GP-ADF and GP-UKF, respectively, the prediction step and update step of the multisensor estimation fusion are provided. In general, it is hard to analyze the performance of the non-parametric fusion methods. Since the Gaussian process fusion methods have analytic mean and covariance, we show that the distributed estimation fusion is equivalent to the centralized estimation fusion with the single sensor cross terms being full column rank. Numerical examples show that the equivalence is satisfied under the condition and the multisensor estimation fusion performs better than the single sensor. We also compare the proposed fusion methods with the RCC-CI [37] algorithm and the convex combination method [43]. In the Gaussian process model setting, the simulation results show that the multisensor estimation fusion methods outperform the RCC-CI algorithm and the convex combination method, as far as the estimation accuracy is concerned.

This article also extends our earlier work [48]. Compared with the conference paper, the main differences are as follows:

- Detailed proofs are provided.
- The enhancement of the multisensor fusion algorithm with GP-UKF is presented.
- Additional set of extensive experiments are carried out.
- The equivalence condition of Proposition 1 is analyzed.
- Comparison between GP-ADF fusion and GP-UKF is given.

The rest of the paper is organized as follows. In Section 2, the problem formulation and the Gaussian process are introduced. In Section 3, the centralized estimation fusion and the distributed estimation fusion methods are presented. In Section 4, simulations are provided to confirm our analysis. Some conclusions are drawn in Section 5.

## 2. Preliminaries

### 2.1. Problem Formulation

**Figure 1:**
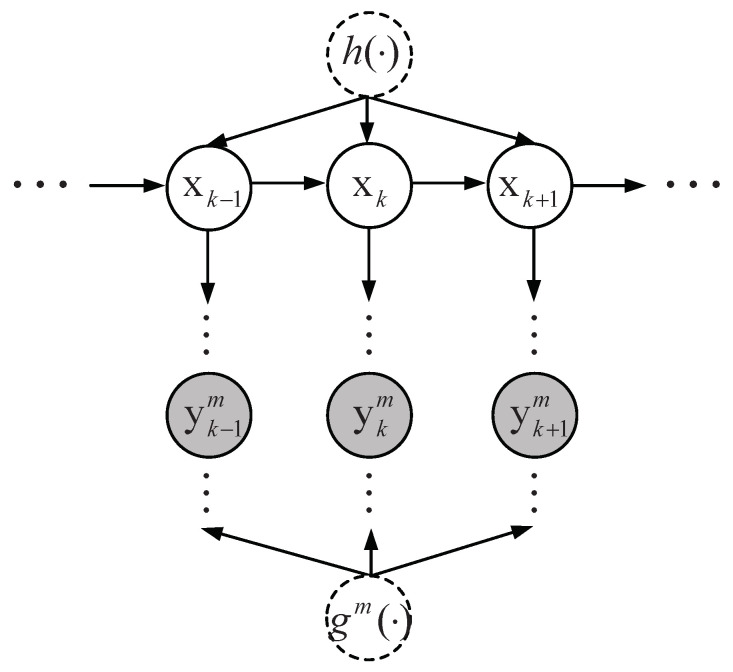
Graphical structure for multisensor nonlinear dynamic systems.

In this paper, we consider the state estimation problem of a nonlinear dynamic system with additive noise and *N* (N≥2) sensor measurements. The multisensor nonlinear dynamic system with state equation and *N* measurement equations (see Figure 1) is described as follows:

(1) $$\begin{array}{cc} x_{k} & =h\left(x_{k-1}\right)+w_{k},\;k=0,1,\ldots , \end{array}$$

(2) $$\begin{array}{cc} y_{k}^{m} & =g^{m}\left(x_{k}\right)+v_{k}^{m},\;m=1,\ldots ,N, \end{array}$$

where $x_{k}\in R^{D}$ is the state of the dynamic system at time *k* and $y_{k}^{m}\in R^{l_{m}}$ is the measurement of the $m_{th}$ sensor at time *k*, m=1,…,N. $h(x_{k})$ is the transition function of the state $x_{k}$ and $g^{m}\left(x_{k}\right)$ is the nonlinear measurement function of $x_{k}$ at the $m_{th}$ sensor. $w_{k}∼N\left(0,Q_{k}\right)$ is a Gaussian system noise and $v_{k}^{m}∼N\left(0,R_{k}^{m}\right)$ is a Gaussian measurement noise, which is independent of each other.

Our goal is to estimate the state from all the available sensor measurement information. Gaussian processes are regarded as the prior of the transition function $h(x_{k-1})$ and the sensor measurement function $g^{m}\left(x_{k}\right)$, m=1,…,N, respectively. Then we make inference about the posterior distribution of the transition function and the sensor measurement function. The Gaussian process model represents a powerful tool to make Bayesian inference about functions [49].

### 2.2. Gaussian Processes

A Gaussian process is defined over functions, which is a generalization of the Gaussian probability distribution [8]. It means that we should consider inference directly in function space. Also, it gives a formal definition, “the Gaussian process is defined a collection of random variables, any finite number of which have a joint Gaussian distribution,” in [8].

Similar to a Gaussian distribution, knowledge of both mean and covariance function can specify a Gaussian process. The mean function m(x) and covariance function (also called a kernel) $k(x,x^{\prime })$ of a Gaussian process f(x) are defined as follows,

$$\begin{array}{cc} m(x) & =E[f(x)],\\ k(x,x^{\prime }) & =E[\left(f\left(x\right)-m\left(x\right)\right)\left(f\left(x^{\prime }\right)-m\left(x^{\prime }\right)\right)], \end{array}$$

where E[·] denotes the expectation. Thus, we write the Gaussian process as

$$\begin{array}{c} f\left(x\right)∼\mathrm{GP}(m\left(x\right),k\left(x,x^{\prime }\right)). \end{array}$$

Unless stated otherwise, a prior mean function is usually assumed to be 0. The choice of the covariance functions depends on the application [14]. In this paper, we employ the most commonly-used squared exponential (SE) kernel in machine learning, which is the prototypical stationary covariance function and useful for modeling particularly smooth functions. It is defined as

(3) $$\begin{array}{cc} & \mathrm{Cov}\left(f\left(x\right),f\left(x^{\prime }\right)\right)=k\left(x,x^{\prime }\right)\\ & \;\;=\alpha^{2}\exp\left\{-\frac{1}{2}{\left(x-x^{\prime }\right)}^{T}\Lambda^{-1}\left(x-x^{\prime }\right)\right\}, \end{array}$$

where parameter $\alpha^{2}$ represents the variance of the function *f* that controls the uncertainty of predictions in areas of less training sample density and parameter Λ is a diagonal matrix of the characteristic length-scales of the SE kernel. Other commonly employed kernel functions can be seen in Reference [8].

A Gaussian process implies a distribution over functions based upon the obtained training data. Assume that we have obtained a set of training data X={X,y}. X and y are made up of multiple samples drawn from the following standard Gaussian process regression model

(4) $$\begin{array}{c} y=f\left(x\right)+\varepsilon ,\;\varepsilon ∼N\left(0,\sigma_{\varepsilon }^{2}\right), \end{array}$$

where $f:R^{D}\to R$ and f(x)∼GP, N denotes the normalized Gaussian probability density function. Note that Gaussian process regression uses the fact that any finite set of training data and testing data of a Gaussian process are jointly Gaussian distributed. Let $\theta =\{\alpha^{2},\Lambda ,\sigma_{\varepsilon }^{2}\}$, called the hyper-parameters of the Gaussian process. Using the evidence maximization method [8,50], we obtain a point estimate

$$\begin{array}{cc} \hat{\theta } & =\;\arg\max_{\theta }\;\log p\left(y|X,\theta \right), \end{array}$$

from the known training data [51]. We can solve the optimization problem with numerical optimization techniques such as conjugate gradient ascent [8,14]. Next, we infer the posterior distribution over function value $f(x_{*})$ from training data for each inputs $x_{*}\in R^{D}$. In addition, the test input $x_{*}$ can be categorized into two classes, relying on whether it is uncertain or not. The corresponding predictive distribution over $f(x_{*})$ will be presented as follows.

(1) **Predictive Distribution over Univariate Function**

Suppose that the training data is X=(X,y)=$\left\{\left(x_{i},y_{i}\right):i=1,\ldots ,n\right\}$, where $x_{i}$ is a *D*-dimensional input vector, $y_{i}$ is a scalar output, and *n* is the number of training data. Next, two cases, deterministic inputs and uncertain inputs, are considered.

● **Deterministic Inputs**

Conditioned on the training data and the deterministic test input $x_{*}$, the predictive distribution over $f(x_{*})$ is a Gaussian with the mean

(5) $$\begin{array}{c} m_{f}\left(x_{*}\right)=E\left[f_{*}\right]=k_{*}^{T}{\left(K+\sigma_{\varepsilon }^{2}I\right)}^{-1}y=k_{*}^{T}\beta , \end{array}$$

and the variance

(6) $$\begin{array}{c} \sigma_{f}^{2}\left(x_{*}\right)={\mathrm{Var}}_{f}\left[f_{*}\right]=k_{**}-k_{*}^{T}{\left(K+\sigma_{\varepsilon }^{2}I\right)}^{-1}k_{*}, \end{array}$$

where ${\mathrm{Var}}_{f}\left[\;\cdot \;\right]$ represents the variance with respect to *f*,

$$\begin{array}{cc} k_{*} & :=k(X,x_{*}),\\ k_{**} & :=k(x_{*},x_{*}),\\ \beta & :={\left(K+\sigma_{\varepsilon }^{2}I\right)}^{-1}y, \end{array}$$

and K is the kernel matrix with each element satisfying $K_{ij}=k\left(x_{i},x_{j}\right)$. The uncertainty of prediction is characterized by the variance $\sigma_{f}^{2}\left(x_{*}\right)$. More details can be seen in Reference [8].

● **Uncertain Inputs**

When the test input is uncertain, namely $x_{*}$ has a probability distribution, the prediction problem is relatively difficult. Let us consider the predictive distribution of $f(x_{*})$ for the uncertain input $x_{*}∼N\left(\mu ,\Sigma \right)$. According to the results in Reference [7] (or reviewing References [52,53]), we introduce the predictive distribution over the function value as

(7) $$\begin{array}{c} p\left(f\left(x_{*}\right)|\mu ,\Sigma \right)=\int p\left(f\left(x_{*}\right)|x_{*}\right)p\left(x_{*}|\mu ,\Sigma \right)dx_{*}, \end{array}$$

where the mean and variance of the distribution $p(f\left(x_{*}\right)|x_{*})$ are provided with Equations (5) and (6), respectively. Based on the conditional expectation and variance formulae, it yields the mean $\mu_{*}$ and variance $\sigma_{*}^{2}$ of the distribution $p(f\left(x_{*}\right)|\mu ,\Sigma )$ in closed form. In particular, we have

(8) $$\begin{array}{cc} \mu_{*} & =E_{x_{*}}\left[E_{f}\left(f\left(x_{*}\right)|x_{*}\right)|\mu ,\Sigma \right]\\ & =E_{x_{*}}\left[m_{f}\left(x_{*}\right)|\mu ,\Sigma \right]\\ & =\int m_{f}\left(x_{*}\right)N\left(x_{*}|\mu ,\Sigma \right)dx_{*}\\ & =\beta^{T}l, \end{array}$$

and

(9) $$\begin{array}{cc} \sigma_{*}^{2} & =E_{x_{*}}\left[m_{f}{\left(x_{*}\right)}^{2}|\mu ,\Sigma \right]+E_{x_{*}}\left[\sigma_{f}^{2}\left(x_{*}\right)|\mu ,\Sigma \right]\\ & \;-E_{x_{*}}{\left[m_{f}\left(x_{*}\right)|\mu ,\Sigma \right]}^{2}\\ \; & =\beta^{T}\tilde{L}\beta +\alpha^{2}-tr\left({\left(K+\sigma_{\varepsilon }^{2}I\right)}^{-1}\tilde{L}\right)-{\left(\mu_{*}\right)}^{2}. \end{array}$$

Note that l in Equation (8) is from $l={\left[l_{1},\ldots ,l_{n}\right]}^{T}$,

$$\begin{array}{cc} l_{j} & =\int k_{f}\left(x_{j},x_{*}\right)p\left(x_{*}\right)dx_{*}=\alpha^{2}{\left|\Sigma \Lambda^{-1}+I\right|}^{-\frac{1}{2}}\\ \; & \;\times \exp\{-\frac{1}{2}{\left(x_{j}-\mu \right)}^{T}{\left(\Sigma +\Lambda \right)}^{-1}\left(x_{j}-\mu \right)\}. \end{array}$$

tr(·) represents the trace in Equation (9) and we have

$$\begin{array}{cc} {\tilde{L}}_{ij} & =\frac{k_{f}\left(x_{i},\mu \right)k_{f}\left(x_{j},\mu \right)}{|2\Sigma \Lambda^{-1}{+I|}^{\frac{1}{2}}}\\ \; & \;\times \exp\{{\left({\tilde{z}}_{ij}-\mu \right)}^{T}{\left(\Sigma +\frac{1}{2}\Lambda \right)}^{-1}\Sigma \Lambda^{-1}\left({\tilde{z}}_{ij}-\mu \right)\}, \end{array}$$

and

$$\begin{array}{c} {\tilde{z}}_{ij}:=\frac{1}{2}\left(x_{i}+x_{j}\right). \end{array}$$

In general, the predictive distribution in Equation (7) cannot be analytically calculated since it leads to a non-Gaussian distribution, if a Gaussian distribution is mapped through a nonlinear function. Using moment-matching method, the distribution $p(f\left(x_{*}\right)|\mu ,\Sigma )$ can be approximated by the Gaussian distribution $N(\mu_{*},\sigma_{*}^{2})$.

(2) **Predictive Distribution over Multivariate Function**

For the model (4), let us turn to the multiple output case that $f:R^{D}\to R^{E}$, f∼GP. Following the results in References [7,51], we need to train *E* Gaussian process regression models independently. The *a*th model is learned from the training data $\left[X,y^{a}\right]$, $y^{a}={\left[y_{1}^{a},\ldots ,y_{n}^{a}\right]}^{T}$, a=1,…,E, where $y_{j}^{a}$ is the *a*th element of $y_{j}$. It implies that any two targeted dimensions are conditionally independent given the input.

For any deterministic input $x_{*}$, the mean and variance of each target dimension are obtained by Equations (5) and (6). The predictive mean of $f(x_{*})$ is a vector stacked by *E* targeted dimension mean, and the corresponding covariance is a diagonal matrix whose diagonal element is the variance of each targeted dimension.

With the uncertain input $x_{*}∼N\left(\mu ,\Sigma \right)$, the predictive mean $\mu_{*}$ of $f(x_{*})$ also is the stacked *E* predictive mean $\mu_{*}^{a}$ given by Equation (8), a=1,…,E. Unlike predicting at deterministic inputs, targeted dimensions are dependent due to the uncertain input. Denote $f_{a}^{*}=f_{a}\left(x_{*}\right)$ and the corresponding predictive covariance matrix is given by

(10) $$\begin{array}{cc} \Sigma_{*} & |\mu ,\Sigma \\ \; & =\left[\begin{array}{ccc} \mathrm{Var}[f_{1}^{*}|\mu ,\Sigma ] & \cdots & \mathrm{Cov}[f_{1}^{*},f_{E}^{*}|\mu ,\Sigma ]\\ \vdots & \ddots & \vdots \\ \mathrm{Cov}[f_{E}^{*},f_{1}^{*}|\mu ,\Sigma ] & \cdots & \mathrm{Var}[f_{E}^{*}|\mu ,\Sigma ] \end{array}\right]. \end{array}$$

It is obvious that the predictive covariance is no longer a diagonal matrix. Using Equation (9), we can obtain the diagonal element of covariance matrix (10). For each a,b∈{1,…,E}, the off-diagonal elements satisfy

$$\begin{array}{c} \mathrm{Cov}\left[f_{a}^{*},f_{b}^{*}|\mu ,\Sigma \right]=E_{f,x_{*}}\left[f_{a}\left(x_{*}\right)f_{b}\left(x_{*}\right)|\mu ,\Sigma \right]-\mu_{*}^{a}\mu_{*}^{b}. \end{array}$$

Given $x_{*}$, $f_{a}\left(x_{*}\right)$ and $f_{b}\left(x_{*}\right)$ are independent, then we have

(11) $$\begin{array}{cc} & E_{f,x_{*}}\left[f_{a}^{*}f_{b}^{*}|\mu ,\Sigma \right]\\ = & \int \int f_{a}^{*}f_{b}^{*}p\left(f_{a},f_{b}|x_{*}\right)p\left(x_{*}|\mu ,\Sigma \right)dfdx_{*}\\ = & \beta_{a}^{T}\int k_{f}^{a}\left(X,x_{*}\right)k_{f}^{b}\left(x_{*},X\right)p\left(x_{*}|\mu ,\Sigma \right)dx_{*}\beta_{b}, \end{array}$$

where $\beta_{a},\beta_{b}$ can be similarly obtained in Equation (5). For notational simplicity, define

$$\begin{array}{cc} L & :=\int k_{f}^{a}\left(X,x_{*}\right)k_{f}^{b}\left(x_{*},X\right)p\left(x_{*}|\mu ,\Sigma \right)dx_{*}, \end{array}$$

where

$$\begin{array}{cc} L_{ij} & =\alpha_{a}^{2}\alpha_{b}^{2}{\left|\left(\Lambda_{a}^{-1}+\Lambda_{b}^{-1}\right)\Sigma +I\right|}^{-\frac{1}{2}}\\ \; & \;\times \exp\{-\frac{1}{2}{\left(x_{i}-x_{j}\right)}^{T}{\left(\Lambda_{a}+\Lambda_{b}\right)}^{-1}\left(x_{i}-x_{j}\right)\}\\ \; & \;\times \exp\{-\frac{1}{2}{\left(z_{ij}-\mu \right)}^{T}R^{-1}\left(z_{ij}-\mu \right)\},\\ R & :={\left(\Lambda_{a}^{-1}+\Lambda_{b}^{-1}\right)}^{-1}+\Sigma ,\\ z_{ij} & :=\Lambda_{b}{\left(\Lambda_{a}+\Lambda_{b}\right)}^{-1}x_{i}+\Lambda_{a}{\left(\Lambda_{a}+\Lambda_{b}\right)}^{-1}x_{j}. \end{array}$$

Thus, we also approximate the distribution $p(f\left(x_{*}\right)|\mu ,\Sigma )$ with the Gaussian distribution $N(\mu_{*},\Sigma_{*})$. More details can be found in Reference [51].

## 3. Multisensor Estimation Fusion

In this section, an essential prerequisite is that the transition function (1) and the measurement functions (2) are either not known or no longer accessible. Thus, we model the latent functions with Gaussian processes. For the *N*-sensor dynamic systems (1) and (2), suppose that we have obtained some training data of the target state and sensor measurement. In the following, we discuss the centralized and distributed estimation fusion methods to estimate the state from a sequence of noisy sensor measurements.

### 3.1. Centralized Estimation Fusion

First, we consider the centralized estimation fusion method. To facilitate fusion, we stack the multisensor measurement information as follows

(12) $$\begin{array}{cc} & y_{k}={\left[{\left(y_{k}^{1}\right)}^{T},\ldots ,{\left(y_{k}^{N}\right)}^{T}\right]}^{T},\\ & g\left(x_{k}\right)={\left[{\left(g^{1}\left(x_{k}\right)\right)}^{T},\ldots ,{\left(g^{N}\left(x_{k}\right)\right)}^{T}\right]}^{T},\\ & v_{k}={\left[{\left(v_{k}^{1}\right)}^{T},\ldots ,{\left(v_{k}^{N}\right)}^{T}\right]}^{T}. \end{array}$$

Thus the dynamic system (1) and (2) can be rewritten as

(13) $$\begin{array}{cc} x_{k} & =h\left(x_{k-1}\right)+w_{k}, \end{array}$$

(14) $$\begin{array}{cc} y_{k} & =g\left(x_{k}\right)+v_{k}, \end{array}$$

where the covariance of the noise $v_{k}$ is given by

$$\begin{array}{cc} \mathrm{Cov}(v_{k})= & \Sigma_{k}=diag\left(\Sigma_{k}^{1},\ldots ,\Sigma_{k}^{N}\right),\\ \mathrm{Cov}(v_{k}^{m})= & \Sigma_{k}^{m},\;m=1,\ldots ,N. \end{array}$$

Considering the Gaussian process dynamic system setup, two Gaussian process models can be trained using evidence maximization. ${\mathrm{GP}}_{h}$ models the mapping $x_{k-1}\mapsto x_{k},\;R^{D}\to R^{D}$ and ${\mathrm{GP}}_{g}$ models the mapping $x_{k}\mapsto y_{k},\;R^{D}\to R^{N*E}$.

As we know, the key of the estimation is to recursively infer the posterior distribution over the state $x_{k}$ of the dynamic system (13)–(14), which are based on all the sensor measurements $y_{1:k}={\left\{y_{\tau }\right\}}_{\tau =1}^{k}$, including the prediction step and the update step. In particular, the prediction step uses the posterior distribution from the previous time step to produce a predictive distribution of the state at the current time step. In the update step, the current predictive distribution is combined with current measurement information to refine the posterior distribution at the current time step. Next, centralized estimation fusion methods with GP-ADF and GP-UKF are presented, respectively.

(1) **Fusion with GP-ADF**

Note that some approximation methods are required to obtain an analytic posterior distribution for the nonlinear dynamic systems. In particular, for the Gaussian process dynamic system, it is easy to make some Gaussian approximation to the posterior distribution. GP-ADF exploits the fact that the true moments of the Gaussian process predictive distribution can be computed in closed form. The predictive distribution is approximated by a Gaussian with the exact predictive mean and covariance [7]. Based on the following lemma, we present the centralized estimation fusion method with GP-ADF.

**Lemma** **1.** *For the Gaussian process dynamic system (13)–(14), the posterior distribution of the state $x_{k}$ can be approximated by the Gaussian distribution $N(\mu_{k}^{e},C_{k}^{e})$, in which the mean $\mu_{k}^{e}$ and covariance $C_{k}^{e}$ are given as [7]:*

(15) $$\begin{array}{cc} & \mu_{k}^{e}=\mu_{k}^{p}+C_{x_{k}y_{k}}{\left(C_{k}^{y}\right)}^{-1}\left(y_{k}-\mu_{k}^{y}\right), \end{array}$$

(16) $$\begin{array}{cc} & C_{k}^{e}=C_{k}^{p}-C_{x_{k}y_{k}}{\left(C_{k}^{y}\right)}^{-1}C_{x_{k}y_{k}}^{T}, \end{array}$$

*where*

(17) $$\begin{array}{cc} \; & \mu_{k}^{p}=E\left[x_{k}|y_{1:k-1}\right],\\ \; & C_{k}^{p}=\mathrm{Cov}\left(x_{k}|y_{1:k-1}\right),\\ & \mu_{k}^{y}=E\left[y_{k}|y_{1:k-1}\right],\\ \; & C_{k}^{y}=\mathrm{Cov}\left(y_{k}|y_{1:k-1}\right),\\ \; & C_{x_{k}y_{k}}=\mathrm{Cov}\left(x_{k},y_{k}|y_{1:k-1}\right). \end{array}$$

**Remark** **1.** Since GP-ADF algorithm [7] is a state estimation method in the single sensor Gaussian process dynamic system, it is applicable to the multisensor system with all the sensor measurements stacked into a vector. Therefore, Equation *(15)* and Equation *(16)* are called the centralized estimation fusion method with GP-ADF here.

Then, let us present the prediction step and update step of the above fusion method in detail.

● **Prediction Step**

First, this step needs to use the previous posterior distribution $p\left(x_{k-1}|y_{1:k-1}\right)\approx N\left(\mu_{k-1}^{e},C_{k-1}^{e}\right)$ to produce a current predictive distribution $p(x_{k}|y_{1:k-1})$. We write the predictive distribution as

$$p\left(x_{k}|y_{1:k-1}\right)=\int p\left(x_{k}|x_{k-1}\right)p\left(x_{k-1}|y_{1:k-1}\right)dx_{k-1},$$

and $p(x_{k}|x_{k-1})$ is a Gaussian distribution due to h∼GP and $w_{k}∼N\left(0,\Sigma_{w_{k}}\right)$. From this, an analogy with Equation (7) yields the mean $\mu_{k}^{p}$ and the variance $C_{k}^{p}$ of the state $x_{k}$ conditioned on the measurement $y_{1:k-1}$. In particular,

(18) $$\begin{array}{cc} \mu_{k}^{p} & =E(x_{k}|y_{1:k-1})\\ \; & =E[h\left(x_{k-1}\right)|y_{1:k-1}]\\ & =E_{x_{k-1}}\left[E_{h}\left(h\left(x_{k-1}\right)|x_{k-1}\right)|y_{1:k-1}\right]. \end{array}$$

$\mu_{k}^{p}$ is a *D*-dimension mean vector and the computation of every target dimension can be seen in Equation (8). The corresponding covariance matrix

(19) $$\begin{array}{cc} C_{k}^{p} & =\mathrm{Cov}\left(h\left(x_{k-1}\right)|y_{1:k-1}\right)+\mathrm{Cov}\left(w_{k}\right)\\ \; & =\Sigma_{h}+\Sigma_{w_{k}}, \end{array}$$

where $\Sigma_{w_{k}}$ is the covariance matrix of transition noise obtained by the evidence maximization method, and the computation of $\Sigma_{h}$ can be seen in Equation (10).

● **Update Step**

Next, we approximate the joint distribution $p(x_{k},y_{k}|y_{1:k-1})$ with a joint Gaussian $N(\mu_{k},C_{k})$, where

$$\mu_{k}=\left[\begin{array}{c} \mu_{k}^{p}\\ \mu_{k}^{y} \end{array}\right],\;C_{k}=\left[\begin{array}{cc} C_{k}^{p} & C_{x_{k}y_{k}}\\ C_{x_{k}y_{k}}^{T} & C_{k}^{y} \end{array}\right].$$

Note that we are not aiming at approximating the distribution $p(x_{k}|y_{1:k})$ directly. To obtain the joint approximation Gaussian distribution, we use the Gaussian distribution $N(\mu_{k}^{y},C_{k}^{y})$ to approximate the distribution $p(y_{k}|y_{1:k-1})$, which can be done in a same way to the prediction step due to

$$p\left(y_{k}|y_{1:k-1}\right)=\int p\left(y_{k}|x_{k}\right)p\left(x_{k}|y_{1:k-1}\right)dx_{k}.$$

On the other hand, the cross term satisfies

$$\begin{array}{cc} C_{x_{k},y_{k}} & =E_{x_{k},g}\left[x_{k}{\left(y_{k}\right)}^{T}|y_{1:k-1}\right]-\mu_{k}^{p}{\left(\mu_{k}^{y}\right)}^{T}. \end{array}$$

For the unknown term $E_{x_{k},g}\left[x_{k}{\left(y_{k}\right)}^{T}|y_{1:k-1}\right]$, we hav

(20) $$\begin{array}{cc} & E_{x_{k},g_{\tilde{a}}}\left[x_{k}y_{k}^{\tilde{a}}|y_{1:k-1}\right]=E_{x_{k},g_{\tilde{a}}}\left[x_{k}g_{\tilde{a}}\left(x_{k}\right)|y_{1:k-1}\right]\\ = & \int x_{k}\left[\sum_{i=1}^{n}\beta_{i}^{\tilde{a}}k_{g^{\tilde{a}}}\left(x_{k},x_{i}\right)\right]p\left(x_{k}\right)dx_{k}\\ = & \sum_{i=1}^{n}\beta_{i}^{\tilde{a}}\int x_{k}c_{1}N\left(x_{k}|x_{i},\Lambda_{\tilde{a}}\right)N\left(x_{k}|\mu_{k}^{p},C_{k}^{p}\right)dx_{k}\\ = & c_{1}{\left(c_{2}\right)}^{-1}\sum_{i=1}^{n}\beta_{i}^{\tilde{a}}\psi \left(x_{i},\mu_{k}^{p}\right),\; \end{array}$$

where $\tilde{a}=1,2,\ldots ,N*E$, $c_{1}^{-1}$ is the normalization constant of the unnormalized SE kernel, $\psi (x_{i},\mu_{k}^{p})$ is the mean of a new Gaussian distribution that is the product of two Gaussian density functions and $c_{2}^{-1}$ is the normalization constant of the new Gaussian distribution. Consequently, from the joint distribution $p(x_{k},y_{k}|y_{1:k-1})$, we obtain the posterior distribution $p\left(x_{k}|y_{1:k}\right)\approx N\left(\mu_{k}^{e},C_{k}^{e}\right)$ with the mean and variance as Equations (15) and (16), respectively.

**Remark** **2.** From Equations *(15)* and *(16)*, we can see that the centralized fusion method with GP-ADF is similar to the unified optimal linear estimation fusion [40,54]. The difference is that the computational way of $\mu_{k}^{p},C_{k}^{p},\mu_{k}^{y}$,$C_{k}^{y}$ and $C_{x_{k}y_{k}}$ and they are mainly based on the training data due to the nonparametric dynamic system model.

(2) **Fusion with GP-UKF**

Following the single sensor GP-UKF (see e.g., References [14,26]), we present the prediction step and update step of the GP-UKF fusion.

● **Prediction Step**

At time k−1, based on the unscented transform [1], we obtain $X_{k-1}$ containing 2D+1 points through the mean $\mu_{k-1}^{e}$ and covariance $C_{k-1}^{e}$. Using the GP prediction model, the transformed set is given by

(21) $$\begin{array}{c} {\bar{X}}_{k}^{\left[i\right]}={\mathrm{GP}}_{h}^{\left[\mu \right]}\left(X_{k-1}^{\left[i\right]}\right),\;for\;i=1,\ldots ,2D+1, \end{array}$$

and the process noise is computed as

(22) $$\begin{array}{c} Q_{k}={\mathrm{GP}}_{h}^{\left[\Sigma \right]}\left(\mu_{k-1}^{e}\right), \end{array}$$

where ${\mathrm{GP}}_{h}^{\left[\mu \right]}$ and ${\mathrm{GP}}_{h}^{\left[\Sigma \right]}$ are the mean and covariance models with respect to the Gaussian process ${\mathrm{GP}}_{h}$. The predictive mean and covariance are

(23) $$\begin{array}{cc} \mu_{k}^{p} & =\sum_{i=1}^{2D+1}W^{\left[i\right]}{\bar{X}}_{k}^{\left[i\right]}, \end{array}$$

(24) $$\begin{array}{cc} C_{k}^{p} & =\sum_{i=1}^{2D+1}W^{\left[i\right]}\left({\bar{X}}_{k}^{\left[i\right]}-\mu_{k}^{p}\right){\left({\bar{X}}_{k}^{\left[i\right]}-\mu_{k}^{p}\right)}^{T}+Q_{k}, \end{array}$$

where $W^{\left[i\right]}$ is the weight generated in the unscented transform. Using the predictive mean $\mu_{k}^{p}$ and covariance $C_{k}^{p}$, we obtain ${\hat{X}}_{k}^{\left[i\right]}$ through the unscented transform. Thus, based on the GP observation model,

(25) $$\begin{array}{c} {\hat{Y}}_{k}^{\left[i\right]}={\mathrm{GP}}_{g}^{\left[\mu \right]}\left({\hat{X}}_{k}^{\left[i\right]}\right)\;for\;i=1,\ldots ,2D+1, \end{array}$$

and the observation noise matrix is determined by

(26) $$\begin{array}{c} R_{k}={\mathrm{GP}}_{g}^{\left[\Sigma \right]}\left({\hat{X}}_{k}^{\left[i\right]}\right). \end{array}$$

Then the predicted observation and innovation covariance are calculated by

(27) $$\begin{array}{cc} {\hat{y}}_{k} & =\sum_{i=1}^{2D+1}W^{\left[i\right]}{\hat{Y}}_{k}^{\left[i\right]}, \end{array}$$

(28) $$\begin{array}{cc} S_{k} & =\sum_{i=1}^{2D+1}W^{\left[i\right]}\left({\hat{Y}}_{k}^{\left[i\right]}-{\hat{y}}_{k}\right){\left({\hat{Y}}_{k}^{\left[i\right]}-{\hat{y}}_{k}\right)}^{T}+R_{k}. \end{array}$$

Meanwhile, the cross covariance is given by

(29) $$\begin{array}{c} C_{x_{k}y_{k}}=\sum_{i=1}^{2D+1}W^{\left[i\right]}\left({\hat{X}}_{k}^{\left[i\right]}-\mu_{k}^{p}\right){\left({\hat{Y}}_{k}^{\left[i\right]}-{\hat{y}}_{k}\right)}^{T} \end{array}$$

● **Update Step**

Finally, the update can be obtained by the following equations:

(30) $$\begin{array}{cc} \mu_{k}^{e} & =\mu_{k}^{p}+C_{x_{k}y_{k}}S_{k}^{-1}\left(y_{k}-{\hat{y}}_{k}\right), \end{array}$$

(31) $$\begin{array}{cc} C_{k}^{e} & =C_{k}^{p}-C_{x_{k}y_{k}}S_{k}^{-1}C_{x_{k}y_{k}}^{T}. \end{array}$$

**Remark** **3.** Note that GP-ADF propagates the full Gaussian density by exploiting specific properties of Gaussian process models [7]. GP-UKF maps the sigma points through the Gaussian process models instead of the parametric functions and the distributions are described by finite-sample approximations. In contrast to GP-UKF, GP-ADF is consistent and moment preserving [7]. In addition GP-EKF requires linearization of the nonlinear prediction and observation models in order to propagate the state and observation, respectively [14]. GP-PF needs to perform one Gaussian process mean and variance computation per particle and has very high computational cost. For more details about these single sensor filtering methods, it can be seen in [7,14], and so forth. Due to the linearization loss of GP-EKF and high computational cost of GP-PF, we do not consider them and only introduce the multisensor estimation fusion with GP-ADF and GP-UKF in this paper.

### 3.2. Distributed Estimation Fusion

In this subsection, we mainly present the distributed estimation fusion with GP-ADF. For the dynamic systems (1) and (2), assume that there is a prior on initial state $x_{0}$ and each local sensor sends its estimation to the fusion center. Thus, the posterior distribution of $m_{th}$ local state estimation is a Gaussian distribution $N(\mu_{k}^{em},C_{k}^{em})$, where $\mu_{k}^{em}$ and $C_{k}^{em}$ are calculated as follows

(32) $$\begin{array}{cc} & \mu_{k}^{em}=\mu_{k}^{pm}+C_{x_{k}y_{k}^{m}}{\left(C_{k}^{ym}\right)}^{-1}\left(y_{k}^{m}-\mu_{k}^{ym}\right), \end{array}$$

(33) $$\begin{array}{cc} & C_{k}^{em}=C_{k}^{pm}-C_{x_{k}y_{k}^{m}}{\left(C_{k}^{ym}\right)}^{-1}C_{x_{k}y_{k}^{m}}^{T}, \end{array}$$

with

(34) $$\begin{array}{cc} \; & \mu_{k}^{pm}=E\left[x_{k}|y_{1:k-1}^{m}\right],\\ \; & C_{k}^{pm}=\mathrm{Cov}\left(x_{k}|y_{1:k-1}^{m}\right),\\ & \mu_{k}^{ym}=E\left[y_{k}^{m}|y_{1:k-1}^{m}\right],\\ \; & C_{k}^{ym}=\mathrm{Cov}\left(y_{k}^{m}|y_{1:k-1}^{m}\right),\\ \; & C_{x_{k}y_{k}^{m}}=\mathrm{Cov}\left(x_{k},y_{k}^{m}|y_{1:k-1}^{m}\right). \end{array}$$

Then, the local posterior mean and covariance are transmitted to the fusion center to yield the posterior distribution of global state estimation.

In general, the centralized estimation fusion method with directly fusing raw data has better fusion performance than the distributed estimation fusion method based on the processed data. However, in some cases, the distributed estimation fusion is equivalent to the centralized estimation fusion. In particular, in what follows, we propose a distributed estimation fusion method and prove that the distributed estimation fusion method is equivalent to the above centralized estimation fusion method when all cross terms $C_{x_{k}y_{k}^{m}}$, m=1,…,N, have full column rank.

**Proposition** **1.** *Assume that all cross terms $C_{x_{k}y_{k}^{m}}$, m=1,…,N are full column rank, the distributed estimation fusion is equivalent to the centralized estimation fusion as follows*

(35) $$\begin{array}{cc} \mu_{k}^{e} & =\mu_{k}^{p}+C_{x_{k}y_{k}}\sum_{m=1}^{N}{\left(C_{k}^{y}\right)}^{-1}\left(m\right)\left(\mu_{k}^{ym}-\mu_{k}^{y}\left(m\right)\right)\\ & \;+C_{x_{k}y_{k}}\sum_{m=1}^{N}{\left(C_{k}^{y}\right)}^{-1}\left(m\right)C_{k}^{ym}C_{x_{k}y_{k}^{m}}^{+}\left(\mu_{k}^{em}-\mu_{k}^{pm}\right), \end{array}$$

*where*

$$\begin{array}{cc} & \mu_{k}^{y}=\left(\mu_{k}^{y}\left(1\right),\ldots ,\mu_{k}^{y}\left(N\right)\right),\\ \; & \mu_{k}^{y}\left(m\right)=E\left(y_{k}^{m}|y_{1:k-1}\right), \end{array}$$

*and*

$${\left(C_{k}^{y}\right)}^{-1}=\left[{\left(C_{k}^{y}\right)}^{-1}\left(1\right),{\left(C_{k}^{y}\right)}^{-1}\left(2\right),\ldots ,{\left(C_{k}^{y}\right)}^{-1}\left(N\right)\right]$$

*is an appropriate partition of matrix ${\left(C_{k}^{y}\right)}^{-1}$ such that Equation *(35)* holds. The superscript “+” stands for pseudoinverse.*

**Proof.**  According to Equation (15), the mean of centralized fused state is given by

(36) $$\begin{array}{cc} \mu_{k}^{e} & =\mu_{k}^{p}+C_{x_{k}y_{k}}{\left(C_{k}^{y}\right)}^{-1}\left(y_{k}-\mu_{k}^{y}\right) \end{array}$$

(37) $$\begin{array}{cc} & =\mu_{k}^{p}-C_{x_{k}y_{k}}{\left(C_{k}^{y}\right)}^{-1}\mu_{k}^{y}+C_{x_{k}y_{k}}{\left(C_{k}^{y}\right)}^{-1}y_{k} \end{array}$$

(38) $$\begin{array}{cc} & =\mu_{k}^{p}-C_{x_{k}y_{k}}{\left(C_{k}^{y}\right)}^{-1}\mu_{k}^{y}+C_{x_{k}y_{k}}\sum_{m=1}^{N}{\left(C_{k}^{y}\right)}^{-1}\left(m\right)y_{k}^{m}. \end{array}$$

Based on the assumption that all $C_{x_{k}y_{k}^{m}}$ have full column rank, we obtain

(39) $$\begin{array}{c} C_{x_{k}y_{k}^{m}}^{+}C_{x_{k}y_{k}^{m}}=I,\;m=1,\ldots ,N. \end{array}$$

Thus, combining Equations (37) and (38) and Equation (39) yields

(40) $$\begin{array}{cc} & C_{x_{k}y_{k}}{\left(C_{k}^{y}\right)}^{-1}y_{k}\\ = & C_{x_{k}y_{k}}\sum_{m=1}^{N}{\left(C_{k}^{y}\right)}^{-1}\left(m\right)y_{k}^{m}\\ = & C_{x_{k}y_{k}}\sum_{m=1}^{N}{\left(C_{k}^{y}\right)}^{-1}\left(m\right)C_{k}^{ym}C_{x_{k}y_{k}^{m}}^{+}C_{x_{k}y_{k}^{m}}{\left(C_{k}^{ym}\right)}^{-1}y_{k}^{m}. \end{array}$$

In order to obtain the centralized estimation mean, that is, fuse the mean of local sensor state estimation at the fusion center, we use Equations (32) and (40) to eliminate $y_{k}$ from Equation (36). From Equation (32), we have

(41) $$\begin{array}{cc} & C_{x_{k}y_{k}^{m}}{\left(C_{k}^{ym}\right)}^{-1}y_{k}^{m}\\ = & \mu_{k}^{em}-\mu_{k}^{pm}+C_{x_{k}y_{k}^{m}}{\left(C_{k}^{ym}\right)}^{-1}\mu_{k}^{ym}. \end{array}$$

Thus, substituting Equation (41) into Equation (40) and recalling Equations (36)–(38), we have

$$\begin{array}{cc} \mu_{k}^{e} & =\mu_{k}^{p}-C_{x_{k}y_{k}}{\left(C_{k}^{y}\right)}^{-1}\mu_{k}^{y}\\ \; & \;+C_{x_{k}y_{k}}\sum_{m=1}^{N}{\left(C_{k}^{y}\right)}^{-1}\left(m\right)C_{k}^{ym}C_{x_{k}y_{k}^{m}}^{+}\\ \; & \;\times (\mu_{k}^{em}-\mu_{k}^{pm}+C_{x_{k}y_{k}^{m}}{\left(C_{k}^{ym}\right)}^{-1}\mu_{k}^{ym})\\ \; & =\mu_{k}^{p}+C_{x_{k}y_{k}}\sum_{m=1}^{N}{\left(C_{k}^{y}\right)}^{-1}\left(m\right)\left(\mu_{k}^{ym}-\mu_{k}^{y}\left(m\right)\right)\\ \; & \;+C_{x_{k}y_{k}}\sum_{m=1}^{N}{\left(C_{k}^{y}\right)}^{-1}\left(m\right)C_{k}^{ym}C_{x_{k}y_{k}^{m}}^{+}\left(\mu_{k}^{em}-\mu_{k}^{pm}\right). \end{array}$$

Therefore, we obtain the state mean of the distributed estimation fusion. □

**Remark** **4.** The proposed distributed estimation fusion formula is equivalent to the centralized estimation fusion, therefore, the two fusion methods have the same fusion performance. However, distributed estimation fusion is more desirable in real applications in terms of reliability, survivability, communication bandwidth and computational burdens. From Equation *(35)*, we can see that $\mu_{k}^{p}$, $C_{k}^{p}$, $\mu_{k}^{y}$, $C_{k}^{y}$, $C_{x_{k}y_{k}}$ can be calculated in advance and the terms $\mu_{k}^{pm}$, $\mu_{k}^{ym}$, $\mu_{k}^{em}$, $C_{k}^{ym}$, $C_{x_{k}y_{k}^{m}}$ need to be transmitted by local sensors in real time.

**Remark** **5.** From the update step *(30)* of the centralized estimation fusion with GP-UKF, the distributed estimation fusion method *(35)* is also suitable for the GP-UKF. Since the detailed description and proof for the distributed GP-UKF fusion are similar to Proposition 1, we omit the results. Note that the distributed GP-UKF fusion is different from the distributed GP-ADF fusion in the prediction step.

## 4. Numerical Examples

In this section, we assess the performance of our fusion methods with two different examples.

### 4.1. 1D Example

**Figure 2:**
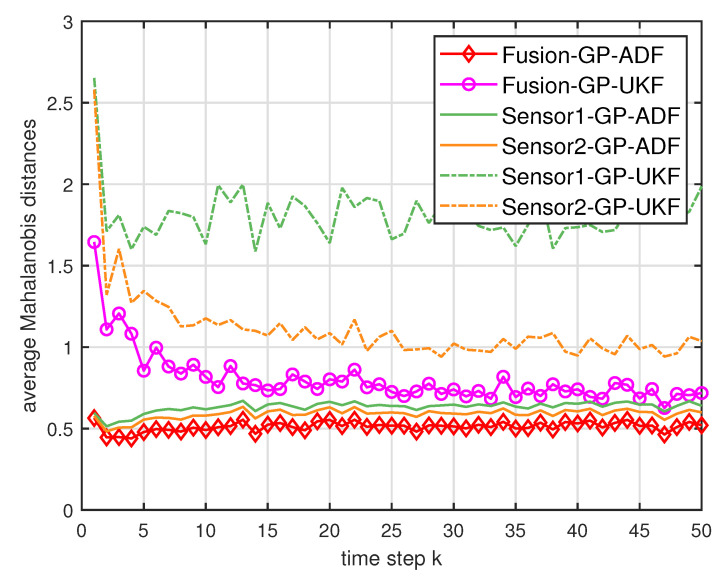
The average Mahalanobis distances of the single sensor and multisensor fusion with $\sigma^{2}=1$.

**Figure 3:**
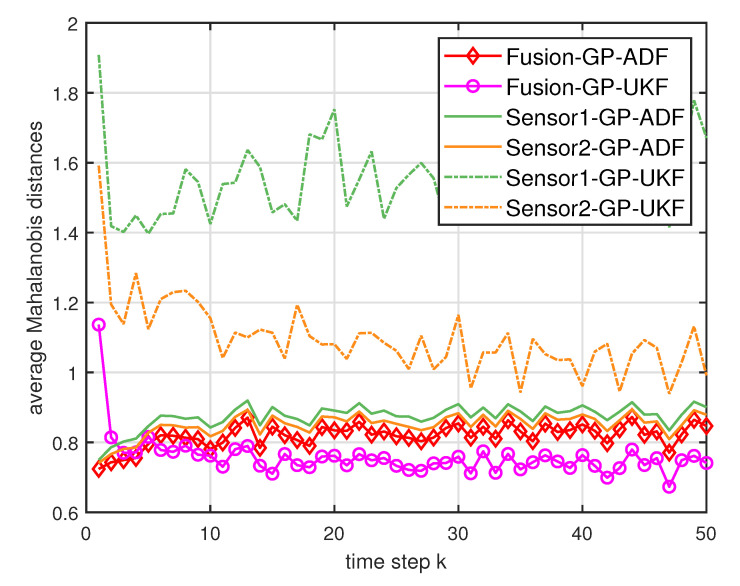
The average Mahalanobis distances of the single sensor and multisensor fusion with $\sigma^{2}=2$.

**Figure 4:**
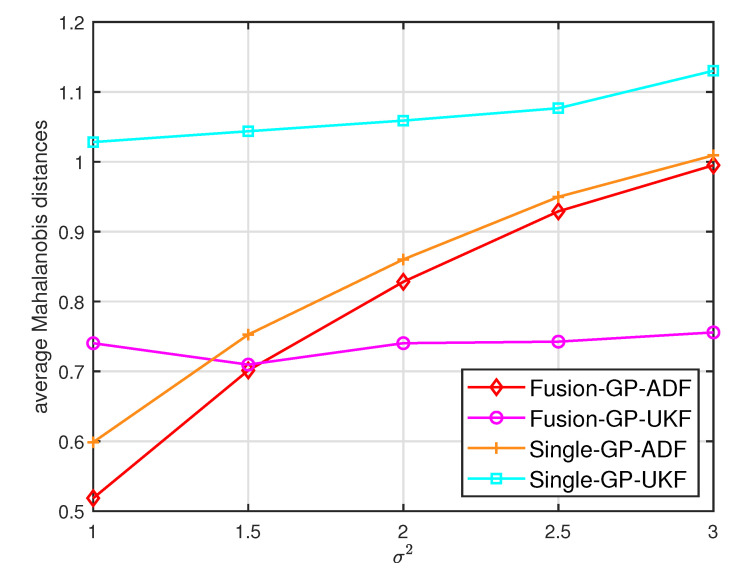
The average Mahalanobis distances for different system noises $\sigma^{2}$.

We consider the nonlinear dynamic system with two sensor measurements as follows:

(42) $$\begin{array}{cc} & x_{k+1}=0.5x_{k}+\frac{25x_{k}}{1+x_{k}^{2}}+w_{k},\;w_{k}∼N\left(0,\sigma^{2}\right) \end{array}$$

(43) $$\begin{array}{cc} & y_{k}^{m}=5\sin\left(2x_{k}+z^{m}\right)+v_{k}^{m},\;v_{k}^{m}∼N\left(0,0.1^{2}\right),m=1,2, \end{array}$$

where $z^{m},m=1,2$ are given by $z^{1}=0$ and $z^{2}=\frac{\pi }{4}$, respectively. This nonlinear system is popularly used in many papers (see References [7,10]) to measure the performance of nonlinear filters. We regard the first 200 samples as the training data and use the rest 50 samples called testing data to test the fusion methods. Assume that the $x_{-200}$ is a Gaussian distribution with prior mean $\mu_{-200}=0$ and variance $\sigma_{-200}^{2}=1.5^{2}$. The estimation performance of the single sensor and multisensor fusion is evaluated by the average Mahalanobis distances that are also used in Reference [7]. The Mahalanobis distance, which is defined between the ground truth and the filtered mean, is as follows:

(44) $$\begin{array}{c} \mathrm{Maha}=\sqrt{{\left(x_{k}-{\hat{x}}_{k}\right)}^{T}{\left(C_{k}^{e}\right)}^{-1}\left(x_{k}-{\hat{x}}_{k}\right)}, \end{array}$$

where $C_{k}^{e}$ is the estimated covariance. For the Mahalanobis distance, lower values indicate better performance [7]. Figure 2 and Figure 3 show the average Mahalanobis distances of the single filter with GP-ADF and GP-UKF, and the fusion methods after 1000 independent runs. Figure 4 shows the average Mahalanobis distances for different system noises.

According to the average Mahalanobis distances in Figure 2 and Figure 3, we can see that the estimation performance of Sensor 2 is better than that of Sensor 1. Furthermore, the multisensor estimation fusion methods perform better than the single sensor filters with GP-ADF and GP-UKF, respectively. Thus the effectiveness and advantages of the multisensor estimation fusion with Gaussian process can be observed. In addition, GP-ADF fusion performs better than GP-UKF fusion for the system noise $\sigma^{2}=1$ and GP-UKF fusion performs better than GP-ADF fusion for the system noise $\sigma^{2}=2$. It means that GP-ADF fusion and GP-UKF fusion have their comparative advantages for different system noises. It suggests that GP-ADF fusion may be a better choice for the small system noise and GP-UKF fusion may be worth considering for the relatively large system noise. From Figure 4, we can see that the proposed fusion methods enjoy better performance for different system noises. It demonstrates the consistence of our fusion methods for different system noises.

### 4.2. 2D Nonlinear Dynamic System

In this subsection, we elaborate the performance of the fusion methods with GP-ADF and GP-UKF, and we consider the example with constant turn motion model in target tracking. The root mean square error (RMSE) is used as the estimation performance measure. It is defined as follows:

(45) $$\begin{array}{c} {\mathrm{RMSE}}_{k}=\sqrt{\frac{1}{M}\sum_{j=1}^{M}{∥x_{k}^{j}-{\hat{x}}_{k}^{j}∥}_{2}^{2}}, \end{array}$$

where *M* is the total number of simulation runs, $x_{k}^{j}$ is the true simulated state for the *j*th simulation, and ${\hat{x}}_{k}^{j}$ is the estimated state value for the *j*th simulation at time *k*, j=1,…,M. The lower the value of the RMSE is, the better the performance of corresponding method is.

#### 4.2.1. Experimental Setup

We consider the constant turn motion model with two sensors as follows:

(46) $$\begin{array}{cc} & x_{k+1}=Fx_{k}+w_{k},\;w_{k}∼N\left(0,Q_{k}\right),\\ & y_{k}^{m}=\left[\begin{array}{c} \sqrt{{\left(x_{k}\left(1\right)-z^{m}\left(1\right)\right)}^{2}+{\left(x_{k}\left(2\right)-z^{m}\left(2\right)\right)}^{2}}\\ arctan\frac{x_{k}\left(2\right)-z^{m}\left(2\right)}{x_{k}\left(1\right)-z^{m}\left(1\right)} \end{array}\right]+v_{k}^{m}, \end{array}$$

(47) $$\begin{array}{cc} & \;v_{k}^{m}∼N\left(0,R_{k}\right),\;\;m=1,2, \end{array}$$

where the state transition matrix F is given by

$$F=\left[\begin{array}{cc} \cos\;\Omega & \sin\;\Omega \\ -\sin\;\Omega & \cos\;\Omega \end{array}\right]$$

with angular velocity Ω, the covariance matrix of transition noise satisfies

$$Q_{k}=\left[\begin{array}{cc} 5 & 0\\ 0 & 5 \end{array}\right],$$

and the covariance matrix of measurement noise is

$$R_{k}=\left[\begin{array}{cc} 5^{2} & 0\\ 0 & {\left(\frac{0.1*\pi }{180}\right)}^{2} \end{array}\right].$$

The sensor positions are given by $z^{1}={\left[-200,200\right]}^{T}$ and $z^{2}={\left[200,-200\right]}^{T}$, respectively.

We use the transition function (46) and measurement functions (47) to simulate a group of data. The values of angular velocity Ω are given by $\Omega =\frac{2\pi }{90}\;\mathrm{rad}/s$ and Ω=0.5rad/s, respectively, which correspond to the small turn motion model and large turn motion model. The first κ samples are regarded as the training data and the rest 50 samples called testing data are used to test the fusion methods. The ${\left\{x_{\tau },y_{\tau }^{1},y_{\tau }^{2}\right\}}_{\tau =-\kappa }^{-1}$ are the true simulated state and two-sensor observations used to train the models. Assume that the $x_{-\kappa }$ is a Gaussian distribution with the prior mean $\mu_{-\kappa }={\left[0,0\right]}^{T}$ and the identity matrix variance $S_{-\kappa }=I$. The initial value of filtering for each sensor is set as the Cartesian coordinate transformation value based on the Polar coordinate observation with a random perturbation for each dimension. We use the random perturbation N(10,1) for the κ=300 training data case and N(20,1) for the κ=30,60 training data cases, respectively. The initial value of fusion filtering is the mean of the initial values of all sensors. Based on the available observation information, the outputs of GP-ADF and GP-UKF for each single sensor are the mean and covariance terms $\mu_{k}^{pm}$, $C_{k}^{pm}$, $\mu_{k}^{em}$, $C_{k}^{em}$, $\mu_{k}^{ym}$,$C_{k}^{ym}$, $C_{x_{k}y_{k}^{m}}$. The distributed fusion methods, the RCC-CI algorithm [37] and the convex combination method [43], are also used as a comparison with our distributed fusion method. The RCC-CI algorithm and the convex combination method directly use the estimated mean and covariance matrix to fuse the estimated results. Our methods take full advantages of the results of the single sensor Gaussian process filters such as the cross term $C_{x_{k}y_{k}^{m}}$ which is easily obtained. Note that all of the fusion methods are based on the local estimates that are distilled from the measurements. Thus, the comparison is fair from the available observation information. The simulation results of the RCC-CI algorithm are based on the YALMIP toolbox [55]. We compare the fusion methods with the RCC-CI algorithm and the convex combination method by RMSE after M=1000 independent simulation runs. Meanwhile, we also compare the computation time of the multisensor estimation fusion methods with the RCC-CI algorithm and the convex combination method. The ratio of full rank, defined as the percentage of full column rank of the cross term $C_{x_{k}y_{k}^{m}}$ for the single sensor filtering at every time step after M=1000 independent runs, is used to test the condition of equivalence. If the ratio of full rank is less than 100%, the condition is broken. The simulations are done under Matlab (MathWorks, Inc., Natick, MA, USA) R2018b with ThinkPad W540.

**Figure 5:**
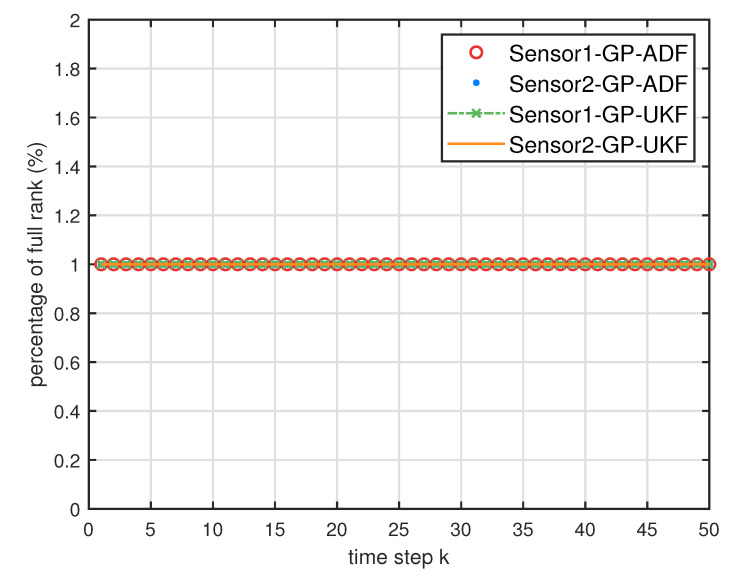
The ratios of full column rank of the cross term $C_{x_{k}y_{k}^{m}}$ in the single sensor case for testing data with κ=300 training data and $\Omega =\frac{2\pi }{90}\;\mathrm{rad}/s,0.5\;\mathrm{rad}/s$.

**Figure 6:**
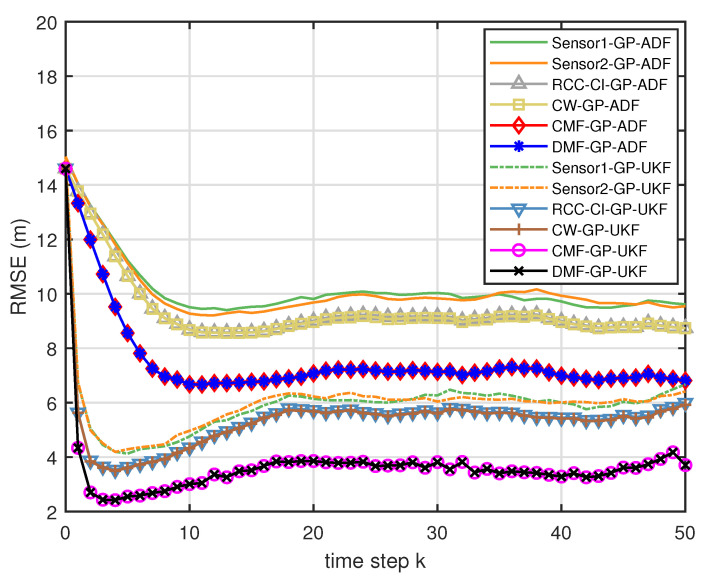
The RMSE for testing data with κ=300 training data and $\Omega =\frac{2\pi }{90}\;\mathrm{rad}/s$.

**Figure 7:**
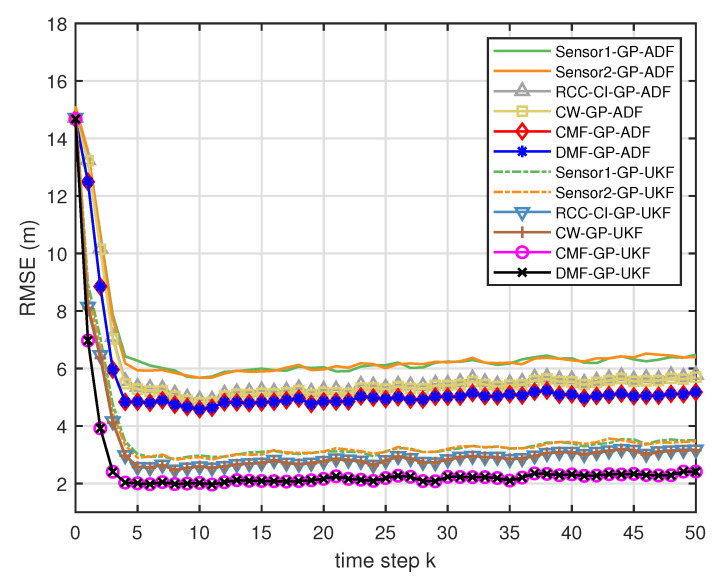
The RMSE for testing data with κ=300 training data and Ω=0.5rad/s.

**Figure 8:**
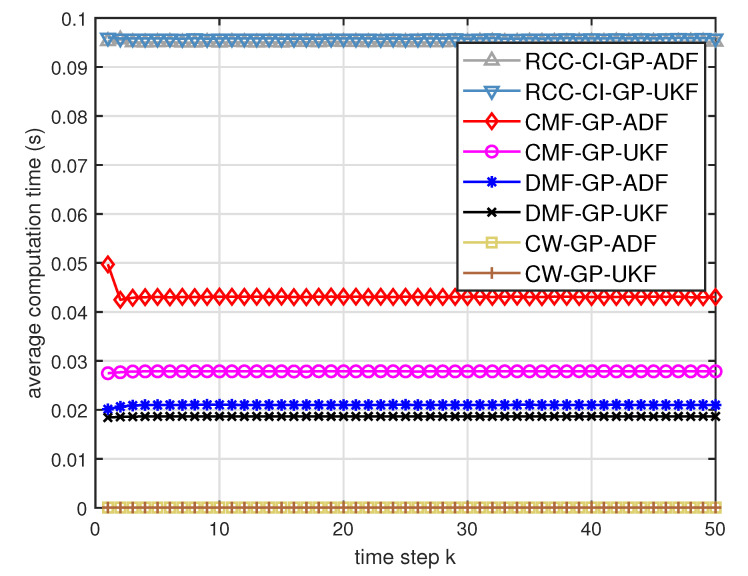
Average computation time of the three fusion methods for testing data with κ=300 training data and $\Omega =\frac{2\pi }{90}\;\mathrm{rad}/s$.

**Figure 9:**
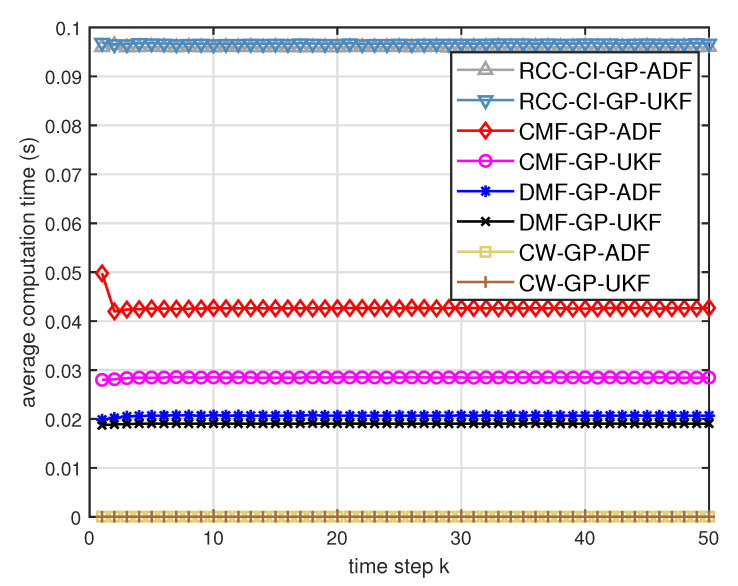
Average computation time of the three fusion methods for testing data with κ=300 training data and Ω=0.5rad/s.

**Figure 10:**
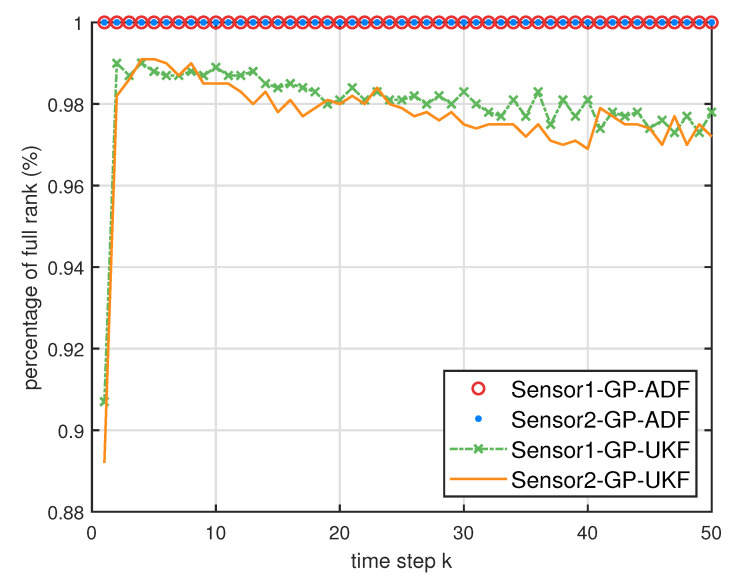
The ratios of full column rank of the cross term $C_{x_{k}y_{k}^{m}}$ in the single sensor case for testing data with κ=30 training data and $\Omega =\frac{2\pi }{90}\;\mathrm{rad}/s$.

**Figure 11:**
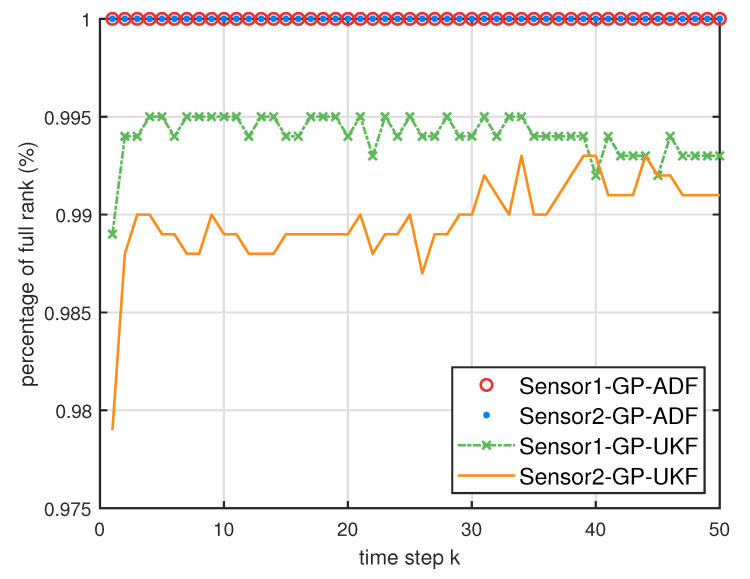
The ratios of full column rank of the cross term $C_{x_{k}y_{k}^{m}}$ in the single sensor case for testing data with κ=60 training data and $\Omega =\frac{2\pi }{90}\;\mathrm{rad}/s$.

**Figure 12:**
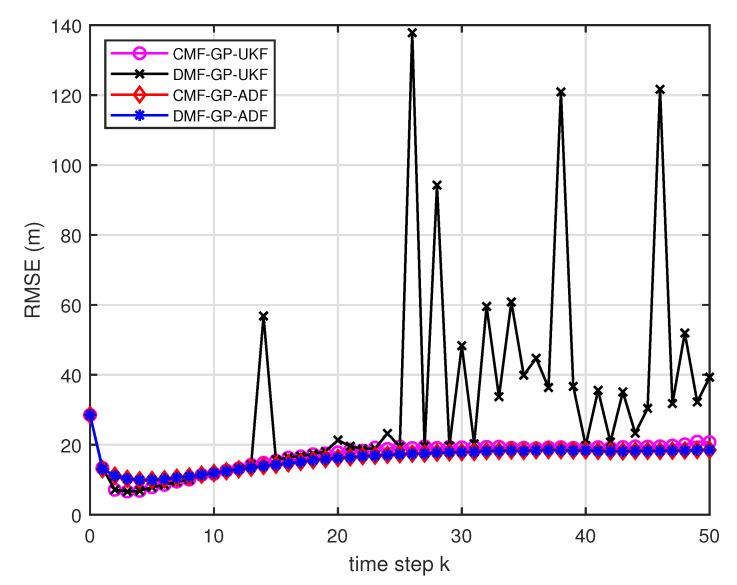
The RMSE for testing data with κ=30 training data and $\Omega =\frac{2\pi }{90}\;\mathrm{rad}/s$.

**Figure 13:**
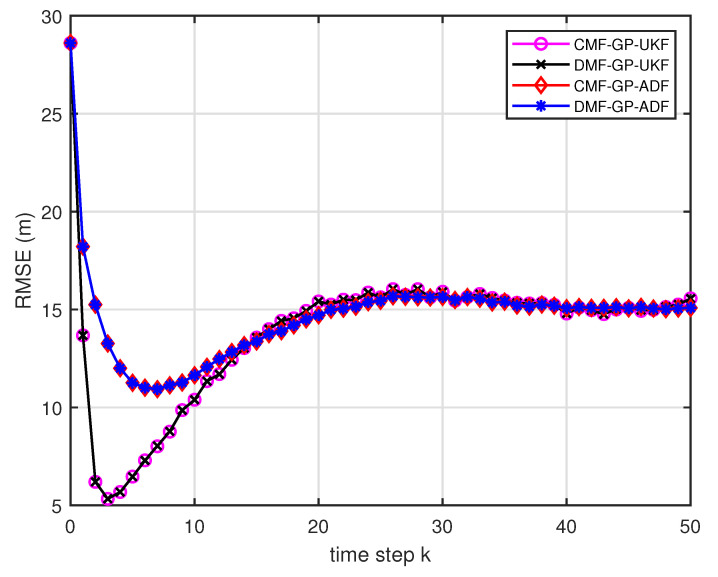
The RMSE for testing data with κ=60 training data and $\Omega =\frac{2\pi }{90}\;\mathrm{rad}/s$.

**Figure 14:**
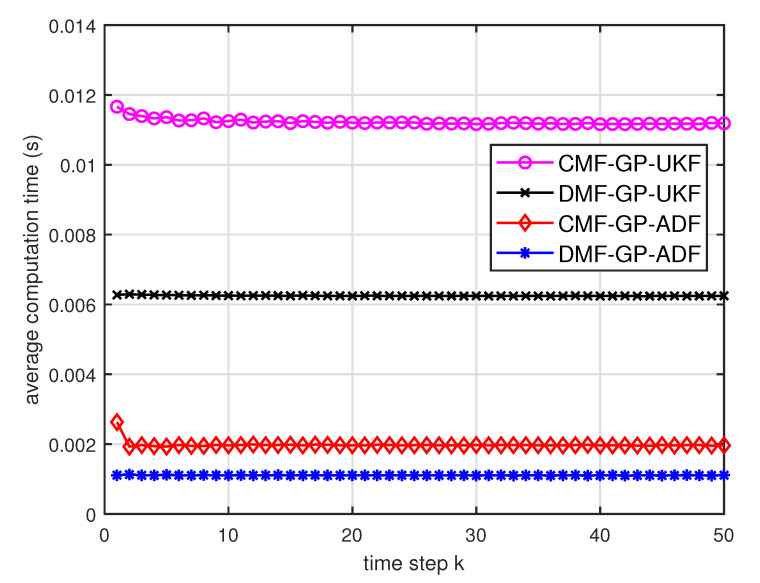
Average computation time of the three fusion methods for testing data with κ=30 training data and $\Omega =\frac{2\pi }{90}\;\mathrm{rad}/s$.

**Figure 15:**
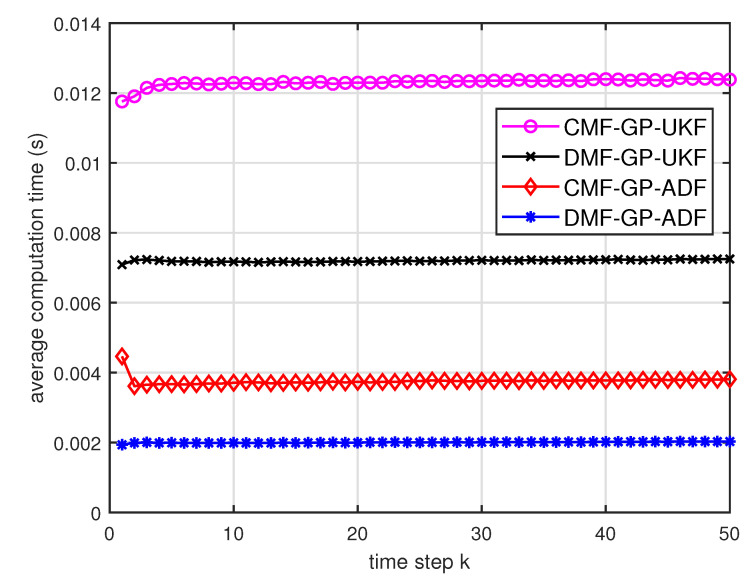
Average computation time of the three fusion methods for testing data with κ=60 training data and $\Omega =\frac{2\pi }{90}\;\mathrm{rad}/s$.

Figure 5 describes the ratios of full column rank in the single sensor case for testing data with κ=300 training data and $\Omega =\frac{2\pi }{90},0.5\;\mathrm{rad}/s$. The RMSEs for $\Omega =\frac{2\pi }{90},0.5\;\mathrm{rad}/s$ in the case of κ=300 training data are depicted in Figure 6 and Figure 7, respectively. The average computation time of these fusion methods for $\Omega =\frac{2\pi }{90},0.5\;\mathrm{rad}/s$ in the case of κ=300 training data after 1000 independent simulation runs are depicted in Figure 8 and Figure 9, respectively. The diamond line represents the RMSE of the centralized estimation fusion with GP-ADF (CMF-GP-ADF) and the star line represents the distributed estimation fusion with GP-ADF (DMF-GP-ADF). The circle line represents the RMSE of the centralized estimation fusion with GP-UKF (CMF-GP-UKF) and the x line represents the distributed estimation fusion with GP-UKF (DMF-GP-UKF). The upper triangle line represents the RMSE of the RCC-CI fusion algorithm with GP-ADF (RCC-CI-GP-ADF) and the lower triangle line represents the RMSE of the RCC-CI fusion algorithm with GP-UKF (RCC-CI-GP-UKF). The square line represents the RMSE of the convex combination method, that is, covariance weight, with GP-ADF (CW-GP-ADF) and the + line represents the RMSE of the convex combination method with GP-UKF (CW-GP-UKF). At the same time, solid line is used for the single sensor GP-ADF filter and dash-dotted line is used for the single GP-UKF filter. Similarly, the ratios of full column rank in the single sensor case for testing data with κ=30,60 training data and $\Omega =\frac{2\pi }{90}\;\mathrm{rad}/s$ are depicted in Figure 10 and Figure 11, respectively. The RMSEs for $\Omega =\frac{2\pi }{90}\mathrm{rad}/s$ in the case of κ=30,60 training data are depicted in Figure 12 and Figure 13, respectively. The average computation time of these fusion methods for testing data with κ=30,60 training data and $\Omega =\frac{2\pi }{90}\;\mathrm{rad}/s$ after 1000 independent simulation runs are depicted in Figure 14 and Figure 15, respectively.

#### 4.2.2. Experimental Analysis

We divide the experimental analysis into two cases, that is, the equivalence condition is satisfied or not. Some phenomena and analysis are described as follows:

**Case 1:** the equivalence condition is satisfied.

- From Figure 5, we can see that the ratios of full rank are all equal to 100%. It means that the cross terms of the single sensor filters are all full column rank for the κ=300 training data case and thus the equivalence condition of centralized fusion and distributed fusion is satisfied in Proposition 1. Meanwhile, from Figure 6 and Figure 7, the RMSE of the distributed estimation fusion is the same as that of the centralized estimation fusion based on GP-ADF and GP-UKF, respectively. It demonstrates the equivalence between the centralized estimation fusion and the distributed estimation fusion in Proposition 1 under the condition of full column rank.
- From Figure 6 and Figure 7, it can be seen that the RMSE of the multisensor estimation fusion method is lower than that of the single sensor filtering. It shows that the multisensor fusion improves the estimation accuracy.
- In addition, the RMSE of multisensor estimation fusion method is lower than that of the RCC-CI algorithm and the convex combination method. It implies the effectiveness of the fusion methods based on Gaussian processes. The possible reason is that our estimation fusion methods extract more extra correlation information, and the RCC-CI algorithm and the convex combination method only use the local estimates with mean and covariance.
- Compared Figure 6 with Figure 7, we can find that our methods do well in the different angular velocity cases, i.e., the small turn motion model and large turn motion model. It confirms that our fusion methods have fine applicability with better performance.
- From Figure 8 and Figure 9, we can find that the computation time of the distributed estimation fusion is less than that of the centralized estimation fusion. It demonstrates the superiority of the distributed estimation fusion under the same fusion performance. The computation time of the proposed fusion methods is much less than that of the RCC-CI algorithm. The possible reason is due to solve a optimization problem for the RCC-CI algorithm. The convex combination method takes the least computation time, since it directly uses the weight combination with covariance.

**Case 2:** the equivalence condition is not satisfied.

- From Figure 10 and Figure 11, it can be seen that the ratios of full rank are both less than 100% for the single sensor GP-UKF case with κ=30 and κ=60 training data. Thus, the equivalence between the centralized and distributed estimation fusion with GP-UKF is broken in Figure 12 and Figure 13, respectively. The reason may be that the Gaussian models are relatively inaccurate with less training data, which can be known from the comparison with Figure 6, Figure 12 and Figure 13 for the same methods. At the same time, the finite-sample approximation of GP-UKF seriously depends on the Gaussian process models and the computation way of the cross terms is the sum about rank-one matrices for GP-UKF. However, the equivalence is still satisfied for the GP-ADF fusion. It implies GP-ADF is more stable than GP-UKF, which is also referred to in Reference [7].
- We can also see that the performance of GP-UKF fusion is better than that of GP-ADF fusion with κ=300 training data from Figure 6 and a little worse with κ=30,60 training data from Figure 12 and Figure 13. Meanwhile, from Figure 8, Figure 14 and Figure 15, the average computation time of GP-UKF fusion is less than that of GP-ADF fusion with κ=300 training data and is contrary with κ=30,60 training data. It may inspire us that the GP-UKF fusion is suitable for the enough training data case and GP-ADF fusion does well in the small number of training data case for the turn motion systems.

In a word, distributed estimation fusion with GP-ADF is more stable, and has a better performance in the case of the small number of training data. If we have enough training data, GP-UKF fusion may be the better choice.

## 5. Conclusions

In the context of this paper, the property matters if the real system does not closely follow idealized models or a parametric model cannot easily be determined for nonlinear systems. A non-parametric method, the Gaussian process, is introduced to learn models from training data, since it takes both model uncertainty and sensor measurement noise into account. In order to estimate the state of the multisensor nonlinear dynamic systems, we have used the Gaussian process models as the prior of the transition and measurement function of the dynamic system. Then the transition function and measurement function have been trained with Gaussian processes, respectively. Based on the Gaussian process models for all available local sensor measurements, we have developed two fusion methods, centralized estimation fusion and distributed estimation fusion, with GP-ADF and GP-UKF, respectively. Taking full advantage of the nature of the Gaussian process, the equivalence between centralized estimation fusion and distributed estimation fusion has been derived under mild conditions. Simulations show that the equivalence is satisfied under given conditions and the multisensor estimation fusion performs better than the single sensor filters. Compared with the RCC-CI algorithm, the multisensor estimation fusion methods not only have higher accuracy, but also require less computation time. The estimation performance of multisensor estimation fusion methods is also better than that of the convex combination method. Future work may involve state initialization, Gaussian process latent variable models without ground truth states, multiple model fusion with different Gaussian processes for maneuvering target tracking and validate the methods with the real sensor data.
